# Worsening of diabetic retinopathy with initiation of an automated insulin delivery system^☆^

**DOI:** 10.1016/j.ajoc.2026.102562

**Published:** 2026-03-13

**Authors:** John M. Nesemann, Devanshi Dove, Frank L. Brodie, Umesh Masharani

**Affiliations:** a Department of Ophthalmology, University of California, San Francisco, USA; b Division of Endocrinology and Metabolism, University of California, San Francisco, USA

**Keywords:** Diabetic retinopathy, Fundus photographs, Insulin pump, Endocrinology

## 1 Case report

**Fig. 1:**
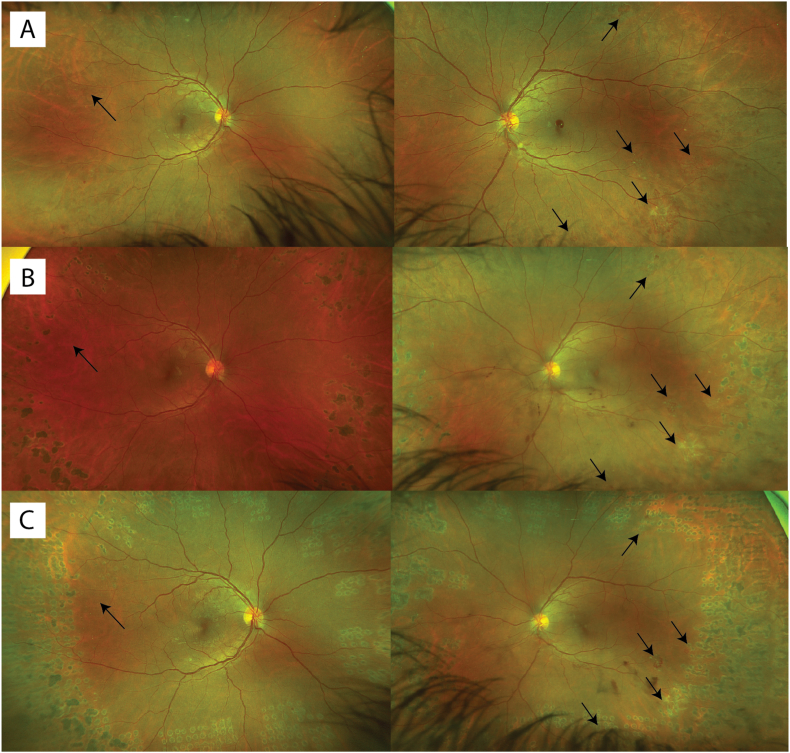
**Fundus photographs in chronological order.** (A) Supratemporal areas of neovascularization in the right eye and supra-and-infra-temporal areas of neovascularization in the left eye. (B) Regressed proliferative diabetic retinopathy prior to iLet pump use. (C) New inferotemporal retinal neovascularization in the left eye with associated pre-retinal hemorrhage after starting the iLet pump.

A 40-year-old man with type 1 diabetes since age 6 struggled to manage his diabetes for many years, often neglecting meal-time boluses and under-dosing insulin due to a fear of hypoglycemia. Medical history was notable for diabetic nephropathy but not hypertension, heart disease, or hypercholesterolemia. Despite using a continuous glucose monitor and continuous subcutaneous insulin infusion (CSII) pump, his HbA1c remained around 11%, leading to mild peripheral neuropathy and non-proliferative diabetic retinopathy by age 32. At 36, he transitioned to an automated insulin delivery system (Tandem control IQ system). This system provided automated correctional insulin doses, lowering his HbA1c to 8% in 6 months. During this time, he developed proliferative diabetic retinopathy in both eyes (Fig. 1A) and underwent pan-retinal photocoagulation (PRP) with regression of retinal neovascularization (Fig. 1B). Ocular coherence tomography at this time did not reveal any diabetic macular edema. Continued difficulty with pre-meal boluses prompted a transition to an automated insulin delivery system, the iLet pump (Beta Bionics, Boston, MA), which had an algorithm accounting for missed meal announcements.^1^ Within two months, his HbA1c improved to 6.7% but was accompanied by new retinal neovascularization and associated pre-retinal hemorrhage (Fig. 1C), for which he underwent additional PRP. His visual acuity remained stable at 20/25 in both eyes.

## 2 Discussion

Rapid lowering of glucose levels can transiently worsen diabetes complications, especially retinopathy. The Steno Diabetes Study and the Diabetes Control and Complications Trial (DCCT) both showed that intensive glycemic control initially exacerbates retinopathy, particularly in patients with pre-existing retinopathy, before conferring long-term benefits.^2^^,^^3^ Theorized mechanisms behind the worsening of retinopathy include changes in retinal blood flow, increased oxidative stress, alterations in vascular endothelial growth factor (VEGF) levels, and osmotic shifts.^2^ As illustrated by this case, automated insulin delivery systems that rapidly lower glucose levels may transiently worsen retinopathy. Whether a slower rate of glycemic improvement is associated with less worsening of retinopathy is uncertain and highlights an area for future research. While rapid glycemic improvement was likely the main driver, other factors, such as blood pressure changes, glycemic variability, nocturnal hypoglycemia, and renal function, may also have contributed to retinopathy progression. The long-term benefits of improved glycemic control, including reduced progression of retinopathy and its complications, outweigh these initial risks, underscoring the importance of offering automated insulin delivery systems to all patients with type 1 diabetes.^3^

## 3 Conclusions

Frequent ophthalmologic monitoring is already recommended during the initial phase of insulin therapy. However, this case suggests individuals starting on automated insulin delivery systems may require even closer monitoring due to the rapid improvement in blood glucose.

## CRediT authorship contribution statement

**John M. Nesemann:** Writing – review & editing, Writing – original draft, Conceptualization. **Devanshi Dove:** Writing – review & editing, Writing – original draft. **Frank L. Brodie:** Writing – review & editing, Writing – original draft, Conceptualization. **Umesh Masharani:** Writing – review & editing, Writing – original draft, Conceptualization.

## Patient consent

Written consent to publish this case has not been obtained. This report does not contain any personal identifying information.

## Authorship

All authors attest that they meet the current ICMJE criteria for Authorship.

## Funding

No funding or grant support

## Declaration of competing interest

The authors declare that they have no known competing financial interests or personal relationships that could have appeared to influence the work reported in this paper.
